# Changes in the prevalence of child marriage in Ethiopia, 2005–2016

**DOI:** 10.1186/s12978-021-01234-4

**Published:** 2022-06-13

**Authors:** Annabel Erulkar

**Affiliations:** Population Council, P.O. Box 25562, Addis Ababa, Ethiopia

**Keywords:** Ethiopia, Child marriage, Trends, Prevalence

## Abstract

**Background:**

Child marriage has powerful implications for a young woman’s reproductive health, education, and personal development as well as the development of communities and nations. Child marriage frequently marks the beginning of a young woman’s sexual activity and early childbearing. As a country where child marriage is common, Ethiopia has placed additional emphasis on addressing child marriage over the past years.

**Methods:**

Using data from Ethiopia Demographic and Health Surveys for 2005, 2011 and 2016, this paper explores trends in child marriage over the last decade in various locations and regions of Ethiopia.

**Results:**

Between 2005 and 2016, the percentage of young Ethiopian women married before age 18 declined from 49 to 40%, a reduction of 18% from 2005 levels. The percentage of women married before age 15 experienced even greater reductions, declining by 26% in the same period. The greatest reductions in child marriage took place in the Addis Ababa, Amhara, and Tigray regions. Over the period, estimates for Oromia and Somali suggest that child marriage has increased in these regions. Notwithstanding recent declines, Afar, Beneshangul-Gumuz, Somali, and Oromia are regions where nearly half or more of all girls are married before age 18.

**Conclusions:**

Nationally, Ethiopia has experienced impressive declines in child marriage over the last decade. However, progress has also been uneven. Trends in the last decade have resulted in a geographical shift in where child marriage is most prevalent. In particular, locations that are challenging in terms of access, including the most remote and hard to reach, pose persistent challenges to those attempting to eradicate the practice. Intensifying efforts in rural areas and underserved regions can facilitate further declines in child marriage in Ethiopia.

## Plain Language Summary

Child marriage has powerful implications for young women’s reproductive health, education, and welfare. Using data from Ethiopia Demographic and Health Surveys for 2005, 2011, and 2016, this paper analyzes subnational trends in child marriage over the last decade.

Between 2005 and 2016, the percentage of Ethiopian women married before age 18 declined from 49 to 40%, a reduction of 18%. The percentage of women married before age 15 experienced even greater reductions, declining by 26% in the same period. The greatest reductions in child marriage took place in Addis Ababa, Amhara, and Tigray regions. Over the same period, estimates for Oromia and Somali regions suggest that child marriage has increased in these regions. Afar, Beneshangul-Gumuz, Somali, and Oromia are regions where more than or nearly half of all girls are married before age 18.

Nationally, Ethiopia has experienced impressive declines in child marriage over the last decade. However, progress has also been uneven, resulting in a geographical shift in where child marriage is most prevalent. Remote and hard-to-reach locations pose persistent challenges to eradicating child marriage. Intensifying efforts in rural areas and underserved regions can achieve the elimination of child marriage in Ethiopia.

## Background

Currently, 650 million women and girls were married before their 18th birthday, with 12 million underage girls married each year [1, 2]. The age at which a woman or girl marries has tremendous implications for her reproductive health trajectory in addition to the negative impacts on her rights, her education, and the development of communities and nations. The practice of child marriage (marriage to someone under age 18) exacerbates the reproductive health vulnerabilities of girls and young women. Child marriage frequently hastens girls into early sexual relations, which are oftentimes unwanted and forced [3]. Likewise, the practice is associated with early childbirth; 90% of adolescent births occur among girls who are already married [4]. Early first births are known to be the riskiest, a risk compounded by the fact that girls who marry as children are often from the poorest communities in the most remote rural locations, where access to quality health care is tenuous, resulting in suboptimal health-seeking behavior [5, 6]. In addition, in many communities of Ethiopia, it is traditional that the first child be born at home, which adds to the risk of early first births associated with child marriage. It is estimated that 25% of known fistula cases are directly attributable to child marriage [7]. Child marriage is also associated with elevated rates of maternal mortality [8], making efforts to eradicate child marriage a critical strategy in improving reproductive health.

Globally, there has been increased attention to the impact of child marriage on girls’ health, their welfare, and the overall development of nations [9]. Over the past decade, UNICEF estimates that 25 million child marriages were averted, 7 million because of expected global declines and 18 million because of increased attention; preventive actions; and interventions by governments, international bodies, and civil society agencies [10]. This decline underscores the potential important contribution that governments and development agencies have in eliminating child marriage. However, the same report highlights that, despite this progress, substantially more effort is required to achieve the Sustainable Development Goal of eliminating the practice by 2030, citing an increasing global burden of child marriage in sub-Saharan Africa. If efforts to prevent child marriage are not intensified, estimates are that 150 million more girls will be married by 2030 [11].

Globally, Ethiopia has the 15th highest rate of child marriage in the world. However, due in part to its large and growing population, the country ranks fifth in the world in terms of the sheer number of child brides, estimated at more than 2.1 million [12]. Furthermore, Ethiopia is among the countries with the highest rate of girls married by age 15 (called under-15 child marriage here), which is arguably the most severe and harmful form of the practice [13].

Ethiopia’s law and policy environment strongly supports the elimination of child marriage. Its Constitution states: “Marriage shall be entered into only with the free and full consent of the intending spouses” (Article 34) [14]. Marriage of a male or female child under the age of 18 is illegal in Ethiopia; the Ethiopia Revised Family Code, issued in 2000, states that “[n]either a man nor a woman who has not attained the full age of eighteen years shall conclude marriage” [15]. Ethiopia has many other policies and action plans that call for the elimination of harmful traditional practices (HTPs), including child marriage, such as the 2013 “National Strategy and Action Plan on HTPs Against Women and Children in Ethiopia” [16].

In 2014, the Ethiopian government committed to eradication of child marriage and female genital mutilation/cutting (FGM/C) at the Girl Summit in London, after which followed a National Girl Summit in Ethiopia in 2015. The Ethiopian government chairs the National Alliance to End Child Marriage and FGM/C, the coordinating body of national efforts to eradicate child marriage, and is 1 of 12 countries implementing the Global Programme to Accelerate Action to End Child Marriage in collaboration with UNICEF and United Nations Population Fund. The attention by nongovernmental organizations (NGOs) to the issue has also expanded and multiplied over the years including programs such as Berhane Hewan (Population Council; 2006–present) [17–19], End Child Marriage Programme: Finote Hiwott (Maxwell Stamp; 2011–2017) [20], TESFA (Care; 2010–2017) [21], and Hiwott Ethiopia [22], all implemented in the Amhara Region.

More recently, in 2019, the Ethiopia Federal Ministry of Women, Children and Youth launched the National Costed Roadmap to End Child Marriage and FGM/C. The roadmap recommends five pillars of action: (1) empowering adolescent girls and families, (2) community engagement, (3) enhancing systems accountability and services, (4) promoting an enabling environment and (5) increasing data and evidence generation and utilization. Importantly, the roadmap highlights not only the harmful effects of child marriage on girls and women but also the economic benefits and reduction in population growth that accrue as a result of eliminating child marriage [23].

This paper explores trends in child marriage in Ethiopia over the past decade. Though available national data are not perfectly aligned with Ethiopia’s intensification of attention to the practice, we use data from the Ethiopia Demographic and Health Surveys (EDHSs) for 2005, 2011, and 2016 to explore changes in the prevalence of child marriage, highlighting where the most and fewest gains have been made in eradicating the practice.

This paper does not explore correlates of child marriage, which have been extensively researched. Rather, we look at trends in different subnational regions of Ethiopia, suggesting direction for where intensifying future efforts and investments in child marriage prevention is warranted. With resources for child marriage programming limited, and the geography and population size of Ethiopia extremely large, the aim of this paper is explicitly to guide policymakers, program managers, and donors as to where progress has been made as well as where substantial levels of child marriage persist. We focus on descriptive data only to demonstrate the magnitude of the practice in various regions and do not examine factors that are correlated to or explain child marriage. Therefore, the aim of the analysis is to make better use of limited programmatic resources in order to geographically focus prevention efforts.

## Methods

Data used for this paper are from EDHSs, which are nationally representative household surveys collecting information from reproductive-age women 15–49 years old. For our analysis, we focus on the last three rounds of EDHS, collected in 2005 [24], 2011 [25], and 2016 [26]. Mini-EDHSs were carried out in 2014 and 2019. However, these surveys do not include sufficient numbers of 20- to 24-year-olds, which is the cohort of women we use in this analysis.

Using data from the 2005, 2011, and 2016 surveys, we present descriptive results that examine the proportion of young women married both during early adolescence, meaning by age 15, and before age 18, which are commonly used standards of expressing child marriage prevalence. Variables were constructed to reflect those married by age 15 and age 18, using marital status and age at marriage/cohabitation variables available in the datasets. The two variables are not mutually exclusive; the variable reflecting those married before age 18 (called under-18 child marriage) also includes girls married before age 15. For analysis, we select the cohort of young women ages 20–24. We did this to account for censored cases and to reflect the youngest cohort of women—and thereby most recent trends—for each round of the survey. Data are weighted using the sample weight available in the datasets. We have not analyzed age at marriage across different age cohorts of Ethiopian women because previous research has demonstrated that reports of age at marriage are subject to reporting errors, especially in older cohorts of women [27].

Using descriptive results, we analyze trends over three successive surveys at the national level, at the regional level, and by urban-rural residence. The percentage change between 2005 and 2011 is calculated using the change in proportions married between 2005 and 2011, divided by 2005 levels of child marriage. In addition, the absolute percentage point change between 2005 and 2011 is presented.

## Results

From 2005 to 2016, Ethiopia experienced overall declines in child marriage that are impressive. Nationally, the percentage of young women married before age 18 was 49% in 2005 and 40% in 2016, an 18% reduction (Table 1). Under 15-child marriage experienced even greater declines, from 19% to 2005 to 14% in 2016, a 26% reduction. Progress in the elimination of child marriage varied between urban and rural areas. Urban areas experienced greater declines in child marriage compared to rural areas. In 2005, the prevalence of women married as children in urban areas was 32%, whereas in 2016, the prevalence was 16%, a 51% reduction. During the same period, prevalence in rural areas declined from 53% to 2005 to 48% in 2016, a 10% reduction. According to estimates from the 2016 EDHS, child marriage is still quite prevalent in rural areas of the country, with nearly half (48%) of young women ages 20–24 having been married as children.

**Table 1:** Percentage of young women aged 20 to 24 married by age 18 and age 15, by year and urban/rural residence or 2005, 2011, and 2016

	Percent 2005 (n = 2844)	Percent 2011 (n = 3022)	Percent 2016 (n = 2903)	Percentage point change 2005–2016	Percent difference 2005–2016
Married by age 18					
Ethiopia (all)	49.1	41.2	40.3	− 8.8	− 17.8
Urban	31.9	21.7	15.7	− 16.2	− 50.8
Rural	53.2	49.0	48.0	− 5.2	− 9.8
Married by age 15					
Ethiopia (all)	19.1	16.4	14.1	− 5.0	− 26.2
Urban	10.5	9.2	2.9	− 7.6	− 72.4
Rural	21.1	19.2	17.7	− 3.4	− 16.1

*EDHS* Ethiopian Demographic and Health Survey

Estimates for percent married by age 18 are inclusive of those married by age 15

From 2005 to 2016, seven of nine regions registered reductions in under-18 child marriage (Table 2). The greatest reductions were in Addis Ababa (52%), Amhara (46%), and Tigray (38%) regions. These regions also had substantial reductions in under-15 child marriage. In particular, the Amhara region declined from an estimated 50% of girls married under the age of 15 in 2005 to 16% of girls married under age 15 in 2016.

**Table 2:** Percentage of young women aged 20 to 24 married by age 18 and age 15, by year and region for 2005, 2011 and 2016

	Percent 2005 (n = 2844)	Percent 2011 (n = 3022)	Percent 2016 (n = 2903)	Percentage point change 2005–2016	Percent change 2005–2016
Married by age 18					
Addis Ababa	16.9	11.8	8.1	− 8.8	− 52.1
Afar	70.0	55.2	66.7	− 3.3	− 4.7
Amhara	79.9	55.6	42.9	− 37.0	− 46.3
Beneshangul-Gumuz	59.3	57.6	50.0	− 9.3	− 15.7
Gambela	62.5	47.1	44.4	− 18.1	− 29.0
Oromia	42.6	40.8	47.7	+ 5.1	+ 12.0
Somali	44.4	52.9	49.4	+ 5.0	+ 11.3
SNNP	31.2	29.7	30.8	− 0.4	− 1.3
Tigray	68.8	43.0	42.8	− 26.0	− 37.8
Married by age 15					
Addis Ababa	6.6	3.0	2.7	− 3.9	− 59.1
Afar	20.0	10.7	18.5	− 1.5	− 7.5
Amhara	50.2	32.8	16.4	− 33.8	− 67.3
Beneshangul-Gumuz	25.9	21.2	15.6	− 10.3	− 39.8
Gambela	14.3	17.6	22.2	+ 7.9	+ 55.2
Oromia	10.3	11.9	17.3	+ 7.0	+ 68.0
Somali	7.4	9.6	9.9	+ 2.5	+ 33.8
SNNP	5.0	8.0	9.6	+ 4.6	+ 92.0
Tigray	26.1	17.0	15.8	− 10.3	− 39.5

*EDHS* Ethiopian Demographic and Health Survey, *SNNP* Southern Nations, Nationalities, and Peoples (region). *Estimates for percent married by age 18 are inclusive of those married by age 15*

Unfortunately, declines in child marriage are not consistent across all regions. Two regions—Oromia and Somali—experienced increases in the proportion of girls married before age 18. From 2005 to 2016, the proportion of young women married before age 18 in Oromia rose from 43 to 48%; in Somali, the proportion rose from 44 to 49%. Based on the estimates, four regions experienced increases in under-15 child marriage from 2005 to 2016: Gambela; Oromia; Somali; and Southern Nations, Nationalities, and Peoples (SNNP).

Despite impressive declines in child marriage over the past 10 years, there are still locations where large proportions of girls are married before their 18th birthday. Afar, Beneshangul-Gumuz, Somali, and Oromia are regions where nearly half or more of all girls are married before age 18. The prevalence of under-15 marriage is also remarkable in several regions, including Gambela (22%), Afar (19%), Oromia (17%), and Amhara (16%), among others.

## Discussion

This study has limitations. The analysis presented here is limited to descriptive data on trends in child marriage in various subnational regions of Ethiopia. This descriptive analysis is explicitly directed toward program managers, policymakers, and donors and provides guidance on where the country has made positive progress and where to geographically focus child marriage prevention and support programs in the future. The analysis does not examine risk factors or correlates of child marriage but attempts to maximize programmatic resources in the country.

Likewise, the analysis does not allow us to confidently draw conclusions on what brought about these changes. Ethiopia’s government has demonstrated a commitment to eradicate child marriage and to increase educational participation; its laws and policies support the elimination of child marriage, and there has been widespread attention by NGOs complemented by a strong evidence base. However, the descriptive results presented here only allow us to surmise that these factors may have contributed to declines in child marriage. Other factors undoubtedly contribute to the trends we witness, such as changing economies, security issues, and underreporting of the practice. Further research is needed to determine why some locations in Ethiopia succeeded in reducing child marriage while others remained unchanged or experienced increases.

Nationally, Ethiopia has experienced impressive declines in child marriage over the last decade. However, progress has been uneven. Urban areas and regions such as Amhara have experienced dramatic declines in the practice. A decade ago, Amhara was known to be the Ethiopian region with the highest rate of child marriage. As a result, Amhara has been the recipient of significant attention to and investments in child marriage prevention, including from NGOs. These efforts appeared to have yielded significant progress toward eradicating the practice, to the extent that Amhara region is now among the regions with relatively lower levels of child marriage.

A decade ago, regions such as Oromia have traditionally not been considered locations where child marriage was widespread, in comparison to other parts of the country. However, failure to focus attention on these locations may have resulted in modest increases in the practice of child marriage over the last 10 years. In addition, the dramatic reductions in regions like Amhara in conjunction with little change in others has meant the locus of the child marriage burden has shifted in Ethiopia to regions such as Afar, Somali, Beneshangul-Gumuz, and Oromia.

These regions, which currently exhibit the highest child marriage prevalence, are all characterized by rural locations that are the hardest to reach and inaccessible, posing challenges to the eradication of child marriage. However, these remote locations are precisely where child marriage tends to persist. Lack of roads, unreliable electricity, low media coverage, weak infrastructure, and limited human resources in remote areas all combine to undermine efforts to create awareness and deliver programs as well as to increase associated costs.

Ethiopia is a country of enormous geographical scale with a large population. Very often, resources are too limited to adequately serve the populations in need. Although datasets such as the EDHS are instrumental in identifying regions where child marriage continues to be prevalent, regional level data are probably still too broad to enable us to efficiently target often limited resources in specific geographical areas. District/woreda-level data would be the most useful for program planners and policymakers to identify the locations with the highest levels of child marriage—the true “hot spots”—and focus investments on prevention and support programs.

## Conclusions

Based on evidence over the past decade, the burden of child marriage is shifting in Ethiopia. In a large country with limited resources, it is critical to use data and evidence to identify locations where the practice is most prevalent. The analysis presented here suggests we need to rethink where to focus child marriage prevention efforts in Ethiopia in order to make further progress in eradication of the practice and maximize returns on these efforts. There is a need to intensify attention in rural areas and underserved regions such as Afar, Somali, Beneshangul-Gumuz, and Oromia. In addition, opportunities to generate and use district-level data will aid in more effective geographic targeting of our efforts.

## Data Availability

All data generated or analyzed during this study are included in this published article. The datasets are available in the 2005, 2011 and 2016 Ethiopia Demographic and Health Survey (EDHS) websites (https://dhsprogram.com/pubs/pdf/FR179/FR179 %5B23June2011 %5D.pdf), (https://dhsprogram.com/pubs/pdf/FR255/FR255.pdf) and (https://dhsprogram.com/pubs/pdf/FR328/FR328.pdf).

## Abbreviations

EDHS: Ethiopia Demographic and Health Surveys
FGM/C: Female genital mutilation/cutting
HTPs: Harmful traditional practices
NGO: Non-Governmental Organization
SNNP: Southern Nations, Nationalities, and Peoples (region)
UNICEF: United Nations Children Fund
